# Remyelination and ageing: Reversing the ravages of time

**DOI:** 10.1177/1352458519884006

**Published:** 2019-11-05

**Authors:** Bjoern Neumann, Michael Segel, Kevin J Chalut, Robin JM Franklin

**Affiliations:** Wellcome Trust-Medical Research Council Cambridge Stem Cell Institute, Jeffrey Cheah Biomedical Centre, University of Cambridge, Cambridge, UK; Wellcome Trust-Medical Research Council Cambridge Stem Cell Institute, Jeffrey Cheah Biomedical Centre, University of Cambridge, Cambridge, UK; Wellcome Trust-Medical Research Council Cambridge Stem Cell Institute, Jeffrey Cheah Biomedical Centre, University of Cambridge, Cambridge, UK; Wellcome Trust-Medical Research Council Cambridge Stem Cell Institute, Jeffrey Cheah Biomedical Centre, University of Cambridge, Cambridge, UK

**Keywords:** Remyelination, stem cell, ageing, oligodendrocyte progenitor cell

## Abstract

Remyelination is a neuroprotective regenerative response to demyelination that restores saltatory conduction and decreases the vulnerability of axons to irreversible degeneration. It is a highly efficient process: however, as with all regenerative processes, its efficiency declines with ageing. Here we argue that this age-related decline in remyelination has a major impact on the natural history of multiple sclerosis (MS), a disease often of several decades’ duration. We describe recent work on (1) how ageing changes the function of oligodendrocyte progenitor cells (OPCs), the cells primarily responsible for generating new myelin-forming oligodendrocytes in remyelination, (2) how these changes are induced by age-related changes in the OPC niche and (3) how these changes can be reversed, thereby opening up the possibility of therapeutically maintaining remyelination efficiency throughout the disease, preserving axonal health and treating the progressive phase of MS.

## Treating progressive MS: a role for remyelination

Since the cause of multiple sclerosis (MS) remains uncertain, the main thrust of MS therapy has been to minimise the consequences of the key pathological events.^1^ A central objective is to prevent axonal loss, which, given that axons do not regenerate in the central nervous system (CNS), will result in cumulative and irreversible functional loss. Thus, in the acute lesion occurring in relapsing-remitting (RR) phase of the disease, the main aim is to suppress the inflammatory response. Achieving this has been one of the great success stories of modern medicine and the advances that have been made in ameliorating the RR stage of the disease have been as impressive as any in neurology therapy in the last 20 years. There are now a wide range of highly effective drugs and treatments (including hematopoietic stem cell therapy).^2,3^ However, none of these have significant impact in treating the progressive phase of the disease, which remains essentially untreatable. There is therefore an urgent medical need to develop effective therapies for progressive MS.^4,5^

The progressive phase of MS is characterised by neuronal and axonal loss. Some of this may result from primary pathology to neurons,^6,7^ a possibility that is still not fully resolved, or, as is the case in the acute lesion in RR, as a consequence of focal inflammation.^8^ However, undoubtedly, much of it occurs because of disruption of the relationship between the axon and the myelin sheath. One of the key functions of the myelin sheath, in addition to conferring the ability to transmit electrical impulses by saltatory conduction, is to preserve the health and integrity of the axon.^9^ The evidence for this first came from transgenic models in which deletions of gene in oligodendrocytes led to secondary degenerative changes in axons.^10,11^ Although not fully understood, the oligodendrocyte, and the myelin sheath it supports, provides metabolic support for the axon via the ‘myelinic channels’, cytoplasmic conduits within the compacted myelin sheath.^12^ This takes the form of delivery of glycolysis products (pyruvate/lactate) via monocarboxylate transporters on the oligodendrocyte to underlying axons, which they use for mitochondrial adenosine triphosphate (ATP) production.^13,14^ Thus, in the absence of a myelin sheath, as occurs after demyelination, the exposed axons are vulnerable to energy deprivation and liable to undergo degeneration from which it may not recover. For this reason, the restoration of new myelin sheaths by remyelination is imperative to preserve axonal integrity and thereby prevent the major pathological change underlying the progressive phase of the disease.^15^ While remyelination occurs as a spontaneous regenerative process early in disease, it is not a sustained process. The failure of regeneration therefore substantially increases the likelihood of axonal degeneration.^16,17^

## How is remyelination achieved?

When oligodendrocytes are lost, the myelin sheaths become detached from the axon and disintegrate, leaving the axon intact. This is the process of demyelination and is a prominent feature of the complex pathology of MS. The default response to demyelination is the spontaneous regenerative process of remyelination, in which new myelin-sheath-forming oligodendrocytes are generated.^18^ The new oligodendrocytes come from an abundant and widespread population of multipotent adult CNS progenitors, called oligodendrocyte progenitor cells (OPCs). In response to injury, the OPCs become activated (a specific change in their gene expression which prepares them for remyelination);^19,20^ migrate and divide, so that they become abundant within the area of demyelination;^21^ and finally differentiate into mature oligodendrocytes that wrap the exposed axons with new myelin sheaths.^22–24^ Although oligodendrocytes do not generate new oligodendrocytes,^25^ it is possible that remyelination may also occur as a result of surviving oligodendrocytes establishing new myelin internodes,^26,27^ although the evidence for this in MS is complicated by difficulties in unambiguously identifying areas of remyelination. Remyelination is a true regenerative process that not only restores the tissue to its pre-lesion architecture, but also restores saltatory conduction and supports axonal survival.^28,29^

## Why does remyelination fail in MS?

Although remyelination can be efficient and widespread in MS, it is not sustained throughout the disease.^30^ There have been many reasons put forward to explain the failure of remyelination in MS.^18,31^ Our contention is that one of the primary and overarching reasons is ageing.

As with all other regenerative processes in mammals, the efficiency of remyelination declines during adulthood.^32,33^ This has been demonstrated experimentally as the time it takes for remyelination to be completed taking progressively longer with increasing age.^34^ With ageing, each step of remyelination becomes slower.^35^ The last phase in which recruited progenitors differentiate is rate limiting, since increasing OPC provision after demyelination in aged animals does not increase the efficiency of remyelination.^36,37^

## Why is the age-dependent decline in remyelination important in MS?

MS is a chronic demyelinating disease, with episodes of focal inflammation and demyelination occurring at intervals throughout the disease, although becoming less frequent with time.^1^ It often starts in young adults in their earlier 20s (although can be much younger), while the age expectancy is only reduced by a few years.^38^ Hence, many individuals affected by MS may have the disease for several decades, meaning that simply by virtue of growing older the ability to remyelinate areas of demyelination declines. An important consequence of this is that demyelinated axons will remain exposed for longer periods and are therefore more vulnerable to irreversible degeneration, a consequence compounded by the likely age-related increase in axonal vulnerability.^39^ There is substantial evidence to support the hypothesis that the age-related failure of remyelination plays a prominent role in the transition from RR disease into progressive disease, characterised by progressive neuronal atrophy and axonal loss. First, a prediction of this hypothesis is that the transition from RR to progressive should occur at around the same age regardless of age of disease onset – and epidemiological evidence exists to support this.^40^ Second, since age primarily affects the differentiation stage of remyelination, then later stages of the disease should be characterised by areas of chronic demyelination that contains undifferentiated cells of the oligodendrocyte lineage. Again, there is a wealth of pathological data to support this – many (although not all) chronically demyelinated lesions contain oligodendrocyte lineage cells that have seemingly failed to differentiate in a timely manner.^41–43^ Third, pathological data indicate that the remyelination capacity declines with disease chronicity.^44^ Fourth, magnetic resonance imaging (MRI) data also support an age-related decrease in remyelination in MS brains.^45^

It follows, therefore, that a logical way of preventing the irreversible axonal loss attributable to age-related remyelination failure is to provide therapies that promote remyelination by rejuvenating endogenous OPCs and their environment. This would provide a treatment for the currently untreatable progressive phase of the disease, for which there is a universally acknowledged urgent medical need.^46^

## What changes occur with ageing that lead to a decline in remyelination efficiency?

Remyelination is ultimately mediated by adult OPCs as they differentiate into new oligodendrocytes. However, there are many cell types present within lesions that contribute to creating an environment conducive to remyelination, including activated astrocytes,^47–49^ electrically active axons,^50^ pericytes,^51^ adaptive immune cells (regulatory T cells)^52^ and innate immune cells (macrophages/microglia).^53–55^ Thus, age effects can be divided into those that are cell-autonomous changes in ageing OPCs or non-cell-autonomous changes that occur in cell types indirectly involved in the formation of new oligodendrocytes. Of the non-cell-autonomous age effects, those on macrophages and microglia, important in remyelination for producing pro-remyelination factors and removing by phagocytosis myelin debris which contains inhibitors of differentiation, have been the most extensively studied. With age, these cells become less efficient at removing and processing myelin debris and alter their gene expression profiles.^56–60^ Astrocytes, which play key roles in remyelination, also change with ageing,^61^ and it is likely that these changes impact their ability to support remyelination, although this has not yet been extensively studied.

## The ageing-induced decline in OPC function

In order to gain a better understating of why remyelination declines cell-autonomous changes in OPCs with ageing, we have developed protocols for isolating OPCs from different aged adult rodents.^62^ This has been a significant breakthrough in understanding the limitations on remyelination since culturing OPCs from aged adult rodents has hitherto proven to be a major technical obstacle. First, we found that while OPCs derived from young adult rats (2–3 months of age – henceforth called young adult OPCs) will differentiate into oligodendrocytes in media commonly used for differentiation, OPCs from aged adult rodents (>12 months – now referred to as aged adult OPCs) hardly differentiate at all. Moreover, while young adult OPCs’ differentiation can be enhanced using thyroid hormone and several other factors that induce differentiation, two of which, benzatropine^63,64^ and 9cis retinoic acid,^65^ are the basis of current remyelination-enhancing clinical trials, these factors fail to induce differentiation in aged adult OPCs. Thus, there is a *central dilemma* in remyelination therapies: as remyelination efficiency declines and the need for remyelination therapies increases, adult OPCs become increasingly less responsive to pro-differentiation agents.

## OPC ageing and the role of mechanosensing

Although adult OPCs undergo functional decline with ageing, it was not known whether this is due to intrinsic, cell-autonomous ageing or due to extrinsic factors such as age-related changes in the OPC niche. To test this, we transplanted aged OPCs, which in their normal environment rarely undergo division, into the neonatal CNS, and found that they proliferated to the same extent as the endogenous neonatal OPCs.^66^ Conversely, young OPCs transplanted into the aged brain cease proliferating. This result strongly suggested that the age-associated changes in OPC function were of extrinsic rather than intrinsic origin. To confirm this, we made substrates from de-cellularised brains from young and aged CNS and seeded onto these OPCs derived from young or old brain. The OPCs acquired the function and age-related transcriptome of the age of the substrate rather than their age of origin. We found, using atomic force microscopy, that a key change in the ageing brain is its increasing stiffness. Since OPCs are known to be mechanosensitive, we tested whether this change was related to OPC ageing by repeating the experiment using chemically inert acrylamide gels, tuned to the stiffness of young or aged brain, and found the same effect. We further went on to show that this stiffness-induced age setting of adult OPCs is mediated by the mechanosensing ion channel, Piezo 1.

## Can the effects of ageing on remyelination be reversed?

Crucial to the success of myelin regenerative therapies as patients age is the question of whether the effects of ageing are reversible. We established that this can be done in a proof-of-principle experiment in 2012 using the model of heterochronic parabiosis, in which lesions in old mice were bathed in factors and cells from young mice.^56^ This resulted in significantly improved remyelination in the aged animals, in part due to the re-modelling of the lesion environment by monocytes derived from the young parabiotic partner. We also showed that remyelination in aged rats could be improved using a single molecule – the retinoid X receptor-γ (RXRγ) agonist 9-*cis* retinoic acid.^65^ We have now generated RNASeq transcriptomic profiles of young and old adult OPCs, which has enabled us to understand better the intrinsic changes occurring in OPCs as they age.^62^ Several pathways change with ageing, including pathways associated with nutrient signalling, which prompted us to examine the effects of calorie restriction (CR) by intermittent fasting, a well-established modulator of ageing, on remyelination in aged animals. We found that fasting had a profound effect, enabling aged animals to remyelinate with the efficiency of young adults. Most intriguingly, we found that this rejuvenation of remyelination can be phenocopied with the 5′ AMP-activated protein kinase (AMPK) agonist and widely prescribed drug, metformin. Moreover, metformin is able to ‘recalibrate’ aged OPCs, reversing the hallmarks of ageing and rendering them responsive to pro-differentiation factors. These results have profound implications for the development of myelin regenerative therapies. They mean that, in combination with metformin, pro-differentiation drugs might now be effective in ageing patients in most need of myelin regenerative therapies.

**Figure 1.**
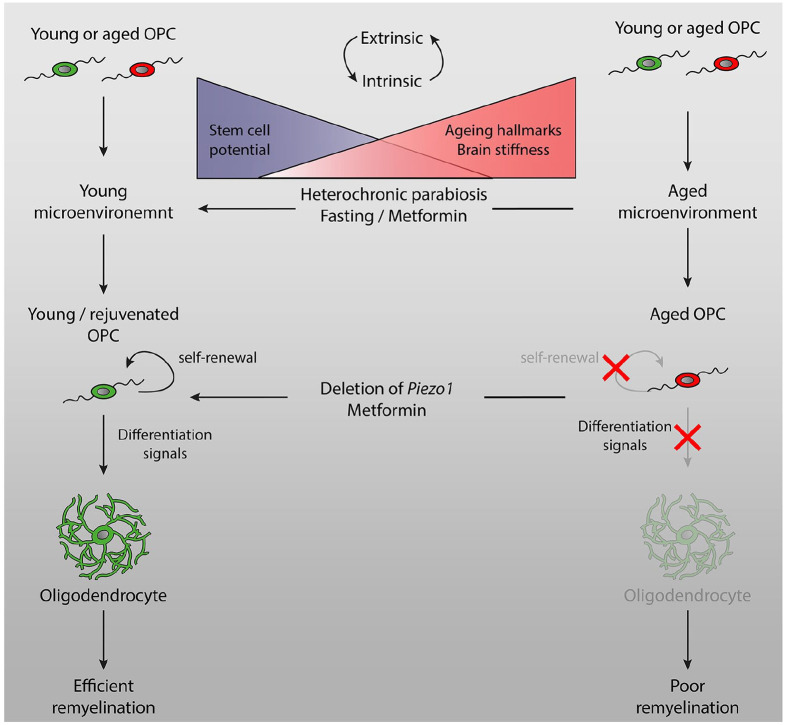
OPCs, like other adult stem cells, undergo functional decline with ageing: they have a diminished ability to self-renew and to differentiate. The dysfunction of aged OPCs is underlined by the acquisition of hallmarks of ageing, like DNA damage and mitochondrial dysfunction. The functional capacity of an OPC is determined by its environment (niche) as OPCs transplanted, irrespective of their own age, function like OPCs of the age of the host tissue (i.e. young or aged OPCs transplanted into a young brain behave like young OPCs). Many biochemical and histological changes that occur in the extracellular environment with ageing have been described, but our recent data demonstrate that the physical properties (stiffness) of the brain play a key role in the ageing process. Interventions, such as heterochronic parabiosis or treatment with metformin, restore a youthful niche and can reinstate the stem cell potential of OPCs and thereby their capacity for remyelination. Alternatively, aged OPCs can be reprogrammed to a more youthful state. The deletion of Piezo 1 prevents OPCs from sensing the stiffness of the niche. Thus, aged OPCs behave as young OPCs that are normally exposed to a soft environment. Therefore, strategies that restore a more youthful environment or that make OPCs impervious to extracellular changes that occur with ageing lead to a functional rejuvenation of OPCs and thereby store the capacity of aged animals for remyelination.

In our studies on the role of mechanosensing by aged OPCs, we found that deleting *Piezo 1* in vitro and in vivo (using a novel OPC-specific CRISPR (clustered regularly interspaced short palindromic repeats)-based gene editing approach delivered systemically using adeno-associated virus (AAV) causes aged OPCs to function as if they were young adult OPCs, providing further evidence that aged OPCs can be rejuvenated and contribute to efficient remyelination.^66^ An interesting but as yet unanswered question is whether CR or CR-mimetics exert their effect, at least in part, by changing the stiffness of the ageing brain. Thus, recent work from our own and other laboratories has made ground-breaking strides in understanding the nature and importance of the age-related decline in remyelination and how, potentially, these may be reversed in a therapeutically meaningful manner (Figure 1).

## Conclusion

Remyelination is a spontaneous regenerative process that naturally follows demyelination. However, as with all regenerative processes in mammals, its efficiency declines with ageing, a phenomenon central to the ageing process itself. This generic feature of the biology of regeneration has important implications for MS, a chronic disease often of several decades’ duration. As an individual progresses through adult life with the disease, their ability to replace lost myelin sheaths decreases, eventually reaching a point where myelin regeneration is too slow to prevent axonal degeneration, and the disease enters the currently untreatable progressive phase. In this article, we argue that a potent way to address the disease progression is to develop therapies that sustain remyelination throughout adult life and reverse the effects of ageing. Central to this goal is to understand the consequences of ageing for the biology of the OPCs. We have shown that the adult OPC acquires all the predictable hallmarks of adult stem cell ageing, which collectively contribute to its declining function and the ability to generate new oligodendrocytes during remyelination. The age-associated decline in function is not due to intrinsic cell ageing but rather is the result of extrinsic changes in the OPC niche and specifically an increase in tissue stiffness. The implications of this is that the age effects on OPCs are not ‘cast in stone’ and that given the correct signals the aged OPC can be reverted to a ‘young’ OPC capable of effective remyelination (Figure 1). This fundamental concept offers enormous hope for the prospects of developing remyelination therapies that will be effective throughout the duration of the disease and that will combat the challenges of treating progressive MS.
